# IL‐38 attenuates myocardial ischemia–reperfusion injury by inhibiting macrophage inflammation

**DOI:** 10.1002/iid3.898

**Published:** 2023-06-14

**Authors:** Yuzhen Wei, Junhui Xing, Xin Su, Xiangrao Li, Xiaofei Yan, Jiangtao Zhao, Hailong Tao

**Affiliations:** ^1^ Department of Cardiology The First Affiliated Hospital of Zhengzhou University Zhengzhou China

**Keywords:** interleukin‐38, macrophages, myocardial apoptosis, myocardial ischemia–reperfusion injury

## Abstract

**Background:**

Reperfusion therapy is the most effective approach to resolve coronary occlusion, but myocardial injury caused by excessive inflammation during myocardial ischemia–reperfusion will also pose a new threat to health. Our prior study revealed the expression pattern of interleukin‐38 (IL‐38) in the peripheral blood serum of patients with ischemic cardiomyopathy and the role of IL‐38 in acute myocardial infarction in mice. However, its role and potential mechanisms in myocardial ischemia/reperfusion injury (MIRI) remain to be determined.

**Methods and Results:**

The left anterior descending artery of C57BL/6 mice was transiently ligated to induce the MIRI model. We found that MIRI induced the expression of endogenous IL‐38, which was mainly produced by locally infiltrating macrophages. Overexpression of IL‐38 in C57BL/6 mice attenuated inflammatory injury and decreased myocardial apoptosis after myocardial ischemia–reperfusion. Furthermore, IL‐38 inhibited lipopolysaccharide‐induced macrophage inflammation in vitro. Cardiomyocytes cocultured with the supernatant of IL‐38‐ and troponin I‐treated macrophages showed a lower rate of apoptosis than controls.

**Conclusions:**

IL‐38 attenuates MIRI by inhibiting macrophage inflammation. This inhibitory effect may be partially achieved by inhibiting the activation of NOD‐like receptor pyrin domain‐related protein 3 inflammasome, resulting in decreased expression of inflammatory factors and reduced cardiomyocyte apoptosis.

## Graphical abstract

Interleukin‐38 attenuates myocardial ischemia–reperfusion injury by inhibiting macrophage inflammation. This inhibitory effect may be partially achieved by inhibiting the activation of NOD‐like receptor pyrin domain‐related protein 3 inflammasome, resulting in decreased expression of inflammatory factors and reduced cardiomyocyte apoptosis.

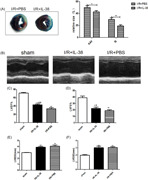

## 1 INTRODUCTION

Reperfusion therapy is the most effective measure to rescue myocardial infarction and preserve cardiac function.^1^ However, myocardial reperfusion injury due to disease pathways and cell death processes has emerged as an important factor affecting the postoperative outcomes of patients.^2^, ^3^ The occurrence and progression of myocardial ischemia–reperfusion (I/R) injury (MIRI) is a complex process regulated by many genes.^4^ Therefore, the mediators and molecular mechanisms involved in MIRI remain under further investigation.

Interleukin‐38 (IL‐38), a member of the IL‐1 family, was discovered in recent years and is encoded by IL1F10.^5^, ^6^ Human IL1F10 shares high sequence homology with receptor antagonists of IL‐1 and IL‐36.^7^, ^8^ Its structure and sequence characteristics determine that it may be involved in the regulation of adaptive and innate immune responses. Many inflammatory diseases driven by well‐defined components of IL‐1 have been reported to be associated with IL‐38 gene polymorphisms.^9^, ^10^, ^11^ Our previous clinical observations showed that acute myocardial infarction (AMI) increased the expression level of IL‐38 in the serum and reperfusion therapy could reduce the level of IL‐38. The correlation analysis suggested that IL‐38 might be a new circulating biomarker for AMI and acute myocardial reperfusion patients.^12^ Our further animal studies showed that IL‐38 ameliorated AMI in mice by regulating phenotypic differentiation of immune cells and expression of related inflammatory factors.^13^ However, the expression and role of IL‐38 after reperfusion in AMI remain to be further elucidated. Macrophages act as system‐wide actors performing numerous tasks, such as wound healing, tissue regeneration, and ventricular remodeling when the heart is in critical condition.^14^ The findings of Ley et al.^15^ demonstrated that IL‐1 family proteins can regulate the production of macrophage cytokines in the context of the microenvironment of the macrophage–apoptotic cell interaction. Moreover, high expression of IL‐38 was detected in the supernatant of some special apoptotic cells, thereby limiting the function of inflammatory macrophage and downstream T‐helper type 17 activation.^16^ These data seem to provide the possibility that IL‐38 targets macrophages to regulate the process of I/R injury.

In this study, we sought to reveal the role and possible mechanisms of IL‐38 in MIRI. Our data suggest that IL‐38 attenuates MIRI by inhibiting macrophage inflammation. This inhibitory effect may be achieved by inhibiting the activation of NOD‐like receptor pyrin domain‐related protein 3 (NLRP3) inflammasome by acting on the IL‐36 receptor, resulting in decreased expression of inflammatory factors and reduced cardiomyocyte apoptosis.

## 2 MATERIALS AND METHODS

### 2.1 Animals

Male C57BL/6 mice were purchased from Beijing HFK Bioscience Co., Ltd. Normal food and water were given to the mice before experiments according to institutional feeding guidelines and suitable room conditions were provided during the feeding cycle in the Animal Care Facility of Zhengzhou University Medical College. All animal studies in this experimental design were in adherence with the Guide for the Care and Use of Laboratory Animals and were approved in advance by the Ethics Committee of Animal Experimentation of Zhengzhou University College.

The experimental animals were divided into different groups and experiments were performed at predetermined time points. The three randomization groups were: (1) sham‐operated group, in which experimental mice underwent operation without left anterior descending (LAD) ligation (sham operation); (2) IL‐38‐treated I/R group, in which mice were intraperitoneally injected with 0.5 μg of recombinant mouse IL‐38 (rIL‐38; Adipogen AG) that were diluted in 200 μL of phosphate‐buffered saline (PBS) 5 min before ligation surgery; (3) PBS‐treated I/R group, in which mice were injected intraperitoneally with the same dose of PBS 5 min before ligation surgery.

### 2.2 Surgical protocol

The MIRI mouse model was induced by transient ligation of the left anterior descending artery as previously described.^17^ Male mice aged 8–10 weeks were anesthetized by intraperitoneal injection of sodium pentobarbital (60 mg/kg, 1%, 5 μL/g), after which the mice were intubated and ventilated by connecting to a rodent respirator and fixed on a specific operating table. A 6‐0 silk suture slipknot was placed around the LAD and ligated for 30 min to induce myocardial infarction after local disinfection and thoracotomy. The slipknot was then released to allow myocardial I/R. In sham operation, the anterior descending artery was not ligated. Tissues were collected at a predetermined point following induction of I/R.

### 2.3 Echocardiography

Mouse cardiac function post‐I/R was assessed on echocardiography using a Vevo 1100 rodent ultrasound machine with a 30 MHz transducer‐phased‐array transducer (Visualsonics). Experimental mice were anesthetized and chest hair was removed before ultrasound examination. Parameters related to mouse cardiac function were calculated using standard formulas by a researcher who was blind to the grouping information, as previously described.^18^ All the mice were anesthetized with sodium pentobarbital (60 mg/kg, 1%, 5 μL/g) before undergoing echocardiography.

### 2.4 Cardiomyocytes

Cardiomyocytes were obtained from suckling mice aged 1–2 days. In brief, heart tissues obtained from suckling mice under aseptic conditions were digested in preprepared 0.05% Trypsin‐EDTA (Gibco) for approximately 30 min on an experimental shaker at 4°C, followed by serial gentle digestions in collagenase type II (Worthington) at 37°C. Cells obtained by digestion as described above were preplated twice in T75 culture flasks to remove fibroblasts by a differential adherent method. Cardiomyocytes obtained from the above procedures were transferred to specific cell culture dishes, followed by supplementing with 15% FCS, 1% streptomycin, 1% penicillin, and 5‐bromo‐2′‐deoxyuridine (100 μmol/L; Sigma). Cardiomyocytes were incubated in a cell incubator at 37°C with 5.0% CO_2_ for subsequent experiments.

### 2.5 Macrophages

RAW264.7 macrophages (ATCC) were cultured in Dulbecco's modified Eagle's medium.^19^ Cells were removed from the plates by gently scraping into fresh medium and then counted using a hemocytometer (BD Bioscience). Macrophage subsets were divided as evenly as possible into different groups: (1) cells with no additional incubation; (2) cells incubated with 500 ng/mL lipopolysaccharide (LPS) for 24 h; (3) cells were incubated with 500 ng/mL LPS and 100 ng/mL IL‐38 for 24 h; (4) macrophages were incubated with 100 ng/mL IL‐38 for 24 h. At the time of harvest, supernatants of each group were collected for detecting the expression of inflammatory factors.

### 2.6 Macrophage/cardiomyocyte coculture

To better mimic the local microenvironment of I/R, we added troponin I (TnI) to assist in the induction of macrophages. RAW264.7 macrophages were transferred to specific culture plates at a density of 2 × 10^5^ cells per well and were stimulated as follows: (1) in the absence of TnI and IL‐38 stimulus or (2) in the presence of 50 ng/mL IL‐38 and 1 μg/mL TnI or (3) in the presence of 1 μg/mL TnI for 24 h. Cells in the (2) and (3) groups had 10 ng/mL LPS added to initiate an immune inflammatory response. At the end of the stimulation time, cells continued to be cultured for 48 h in a new medium. At the time of harvest, the supernatant of each group was collected to detect the expression of inflammatory factors.

The second and third groups of supernatant were used for coculture with cardiomyocytes. The mixed cells were cultured in a thermostatic cell incubator (37°C) under hypoxic conditions (1% O_2_, 5% CO_2_, and 94% N_2_) for 6 h, and then transferred to the normoxia incubator for 6 h to undergo reoxygenation. Cells without anaerobic conditions served as a control.

### 2.7 Measurement of apoptosis

Cardiomyocyte apoptosis was detected by flow cytometry using Annexin V and PI fluorescein staining kit (eBioscience). Cultured cardiomyocytes were collected by centrifugal sedimentation, and then incubated with antibodies following the staining kit instructions. The apoptosis rate was evaluated by Flow Cytometry (FACS Calibur; BD Immunocytometry Systems) and analyzed by Flowjo software.

### 2.8 TUNEL assay

Terminal deoxynucleotidyl transferase dUTP nick‐end labeling (TUNEL) apoptosis detection kit (Roche Diagnostics GmbH) was used to detect myocardial apoptosis. Heart sections of tissue or cultured cardiomyocytes on coverslips were fixed in 4% paraformaldehyde and permeabilized with 0.1% Triton X‐100 according to the instructions. To visualize apoptotic cardiomyocytes, the above‐treated glass slides were incubated with the TUNEL reaction mixture. Total cell nuclei were stained with 4′,6‐diamidino‐2‐phenylindole. Three to five random fields in each sample were examined by a technician blinded to experimental treatment information.

### 2.9 Measurement of myocardial infarct size

Injured hearts were obtained from I/R mice, which were reanesthetized and killed at the end of a 24‐h reperfusion. The thoracic cavity of the anesthetized mice was accessed and Evans blue (Sigma‐Aldrich) was injected into the cardiac apex. Then, the ischemic and nonischemic areas of the heart were colored differently.

The left ventricle was isolated and evenly cut into 1‐mm‐thick transverse slices. The heart sections were infiltrated with PBS and then incubated in 1% TTC (2, 3, 5‐triphenyl tetrazolium chloride; Sigma‐Aldrich) for 40 min at 37°C to differentiate the infarcted heart regions, and then the slices were fixed with 4% formaldehyde. Image‐Pro Plus 6.0 was used to measure the areas at risk and infarct size.

### 2.10 Western blot analysis

The protein expression levels of target genes were detected by western blot. Total proteins were extracted from heart tissues or cultured cells using RIPA lysis buffer (Beyotime) according to instructions provided with the kit and protein concentrations were measured using a BCA Protein Assay Kit (Pierce Biotechnology Inc.). Antibodies used in this study are as follows: glyceraldehyde 3‐phosphate dehydrogenase (GAPDH) (Immunoway), p65NF‐κB (R&D Systems), NLRP3 (Cell Signaling Technology), and IL‐38 (R&D Systems). Equal amounts of denatured protein samples were separated on 10% sodium dodecyl sulfate‐polyacrylamide gel electrophoresis prepared in advance for about 2 h and then were transferred to polyvinylidene difluoride membranes, followed by blocking with 5% nonfat milk for 2 h and incubated with the indicated primary antibodies at 4°C overnight. After incubation with horseradish peroxidase‐conjugated secondary antibodies for about 2 h at room temperature, membranes were treated with ECL reagents (Thermo Scientific). GAPDH was used as the loading control to normalize comparisons, and data were analyzed using a densitometer.

### 2.11 Quantitative real‐time polymerase chain reaction (PCR) analysis

Total RNA was extracted from cardiac tissues or myocardial cells using Trizol (Invitrogen) and was reverse transcribed into complementary DNA using the PrimeScript RT reagent kit (Takara Biotechnology). The messenger RNA (mRNA) levels of the target genes were quantified using SYBR Green Master Mix (Takara Biotechnology) with an Applied Biosystems 7500 Real‐Time PCR system (Bio‐Rad). mRNA expression levels were analyzed by normalizing to GAPDH using the relative threshold cycle method.

### 2.12 Cytokine detection

The levels of cytokines (IL‐38, IL‐6, tumor necrosis factor‐α [TNF‐α], IL‐1β and IL‐10) were detected using ELISA (enzyme‐linked immunosorbent assay) kits (R&D Systems) according to the instructions. Data were analyzed based on the absorbance of the samples read by a microplate reader.

### 2.13 Immunohistochemistry and immunofluorescence

Heart tissues obtained from I/R mice were embedded in paraffin after fixation in 4% paraformaldehyde. Heart sections (4 μm) were processed as previously described and stained with hematoxylin–eosin (HE), anti‐myeloperoxidase (MPO; Abbiotec), and inducible nitric oxide synthase (INOS) (eBioscience). Opositive cells and case areas were observed by a microscope. Heart tissue obtained from mice reperfused for 24 h was used for immunofluorescence colocalization to analyze IL‐38‐expressing cells after I/R. Antibodies used for immunofluorescence were as follows: CD3 (1:100; BioLegend), CD68 (1:100; Santa Cruz Biotechnology), α‐actinin (1:100; Abcam), and vimentin (1:100; BioLegend). The average number of positive cells in the infarct area was used for comparative analysis.

### 2.14 Statistical analysis

Data were presented as means ± SEM. Student's *t*‐test was used to compare the differences between two groups and one‐way analysis of variance was used to analyze the differences among multiple groups, followed by Holm–Sidak test. All data were analyzed using SPSS 13.0 (SPSS), and *p* < .05 was considered statistically significant.

## 3 RESULTS

### 3.1 IL‐38 were induced by reperfusion injury

**Figure 1:**
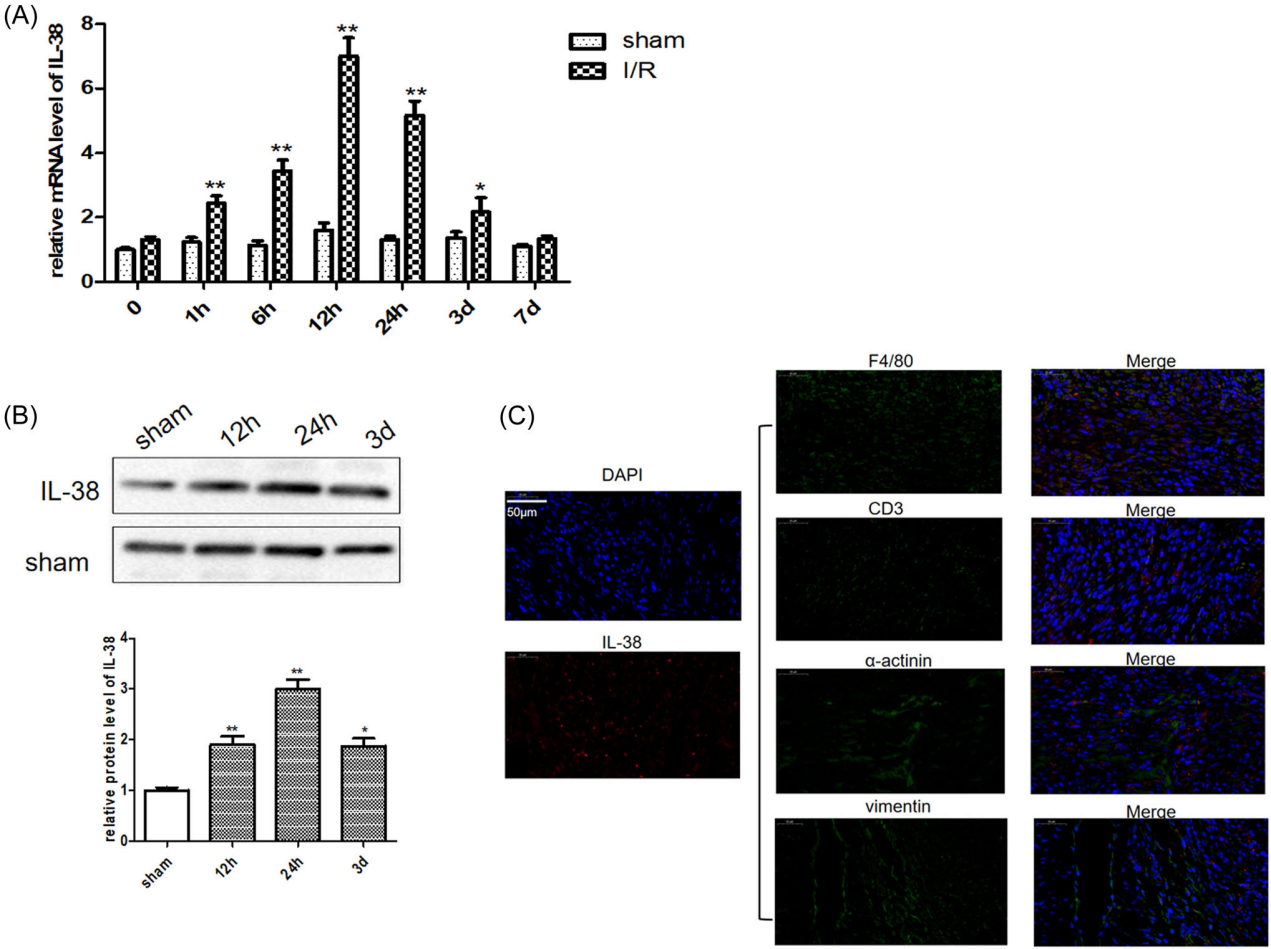
Reperfusion injury stimulated interleukin‐38 (IL‐38) expression. (A) Relative messenger RNA (mRNA) expression of IL‐38 in the border zone of infarcted hearts in C57BL/6 at different time points after ischemia/reperfusion (I/R). (B) Representative image of a western blot for IL‐38 protein expression levels in infarcted hearts in C57BL/6 at different time points after I/R. (C) Representative images of infarcted heart sections (obtained from I/R mice on Day 1) stained with antibodies against F4/80 (green), CD3 (green), α‐actinin (green), vimentin (green), and the cytokine IL‐38 (red), and a nuclear stain (blue). DAPI, 4′,6‐diamidino‐2‐phenylindole. Scale bar: 50 μm; magnification: ×200 (each time point *n* = 8). *p < .05 vs. sham; **p < .01 vs. sham.

Although IL‐38 has been reported to be expressed in various immune organs, its expression in heart disease is rarely reported. To reveal the expression of IL‐38 after reperfusion injury, we examined the mRNA and protein expression levels of IL‐38 in the ischemic myocardium of MIRI mice. As shown in Figure 1A, the expression level of IL‐38 mRNA in the injured heart was increased within 1 h, peaked at 12 h, and remained elevated but with a declining trend for up to 7 days after reperfusion. Similarly, the expression level of IL‐38 protein was also upregulated, as shown in Figure 1B.

IL‐38 has been reported to be secreted by a variety of immune and inflammatory cells. To determine which cells in the reperfusion‐injured heart are responsible for IL‐38 secretion, we performed immunofluorescence colocalization of IL‐38 and major cell types involved in MIRI development. As shown in Figure 1C, most of the IL‐38 signal was detected in F4/80^+^ macrophages. In addition, IL‐38 was detected in α‐actinin^+^ cardiomyocytes, with the lowest expression in CD3^+^ cells and Vimentin^+^ myocardial fibroblasts.

### 3.2 IL‐38 improves the cardiac function of MIRI mice

**Figure 2:**
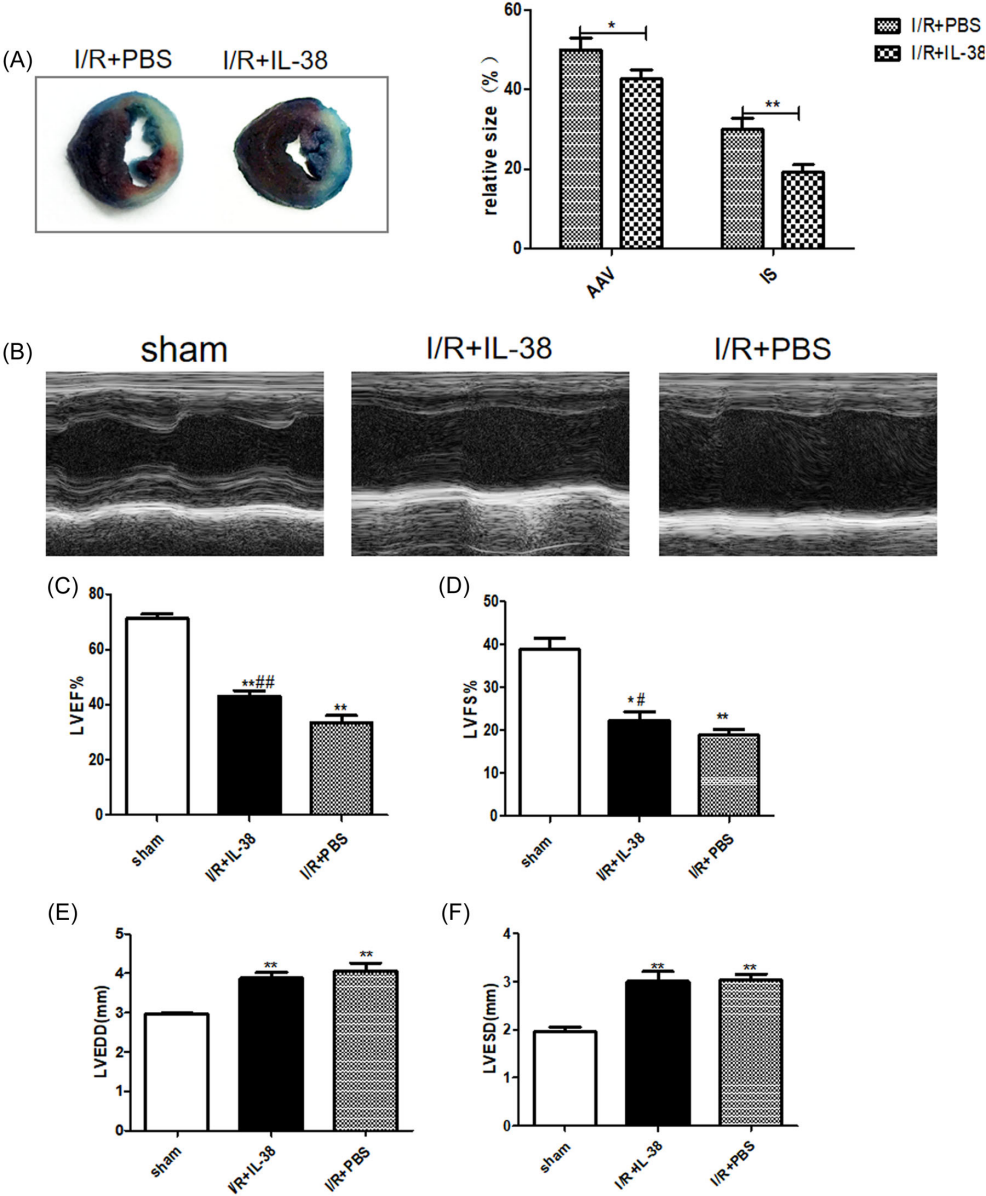
Interleukin‐38 (IL‐38) improves left ventricular function after ischemia/reperfusion (I/R). (A) Representative images of Evans blue and 2, 3, 5‐triphenyl tetrazolium chloride (TTC) staining of myocardium slices and calculation of area at risk and infarct size (relative to left ventricular [LV] area) on Day 1 after I/R. (B) Representative M‐mode echocardiography images of the left ventricle on Day 1 after I/R in different groups as indicated above. (B–E) LV ejection fraction (LVEF), LV fractional shortening (LVFS), LV end‐diastolic diameter (LVEDD), and LV end‐systolic diameter (LVESD) on Day 1 after I/R (each group *n* = 8). *p < .05 vs. sham; **p < .01 vs. sham; ^#^ *p* < .05 vs. I/R + phosphate‐buffered saline [PBS]; ^##^ *p* < .01 vs. I/R + PBS.

To determine the role of IL‐38 in MIRI, we used recombinant mouse IL‐38 (rIL‐38) to intervene in I/R model. Myocardial infarct size and the area at risk of MIRI mice were demonstrated by Evans blue and TTC staining. As shown in Figure 2A, MIRI mice treated with rIL‐38 showed reduced myocardial infarct size and risk area compared to mice receiving PBS. We then calculated left ventricular function‐related indexes. As shown in Figure 2B, rIL‐38‐treated mice showed a higher left ventricular ejection fraction and left ventricular fractional shortening compared to PBS‐treated mice. Correspondingly, left ventricular end‐diastolic diameter was smaller in rIL‐38‐treated mice, although there was no significant statistical significance in left ventricular end‐systolic diameter. As expected, the results of these experiments collectively indicate that IL‐38 plays a protective role in MIRI.

### 3.3 IL‐38 inhibits inflammatory cells infiltration

**Figure 3:**
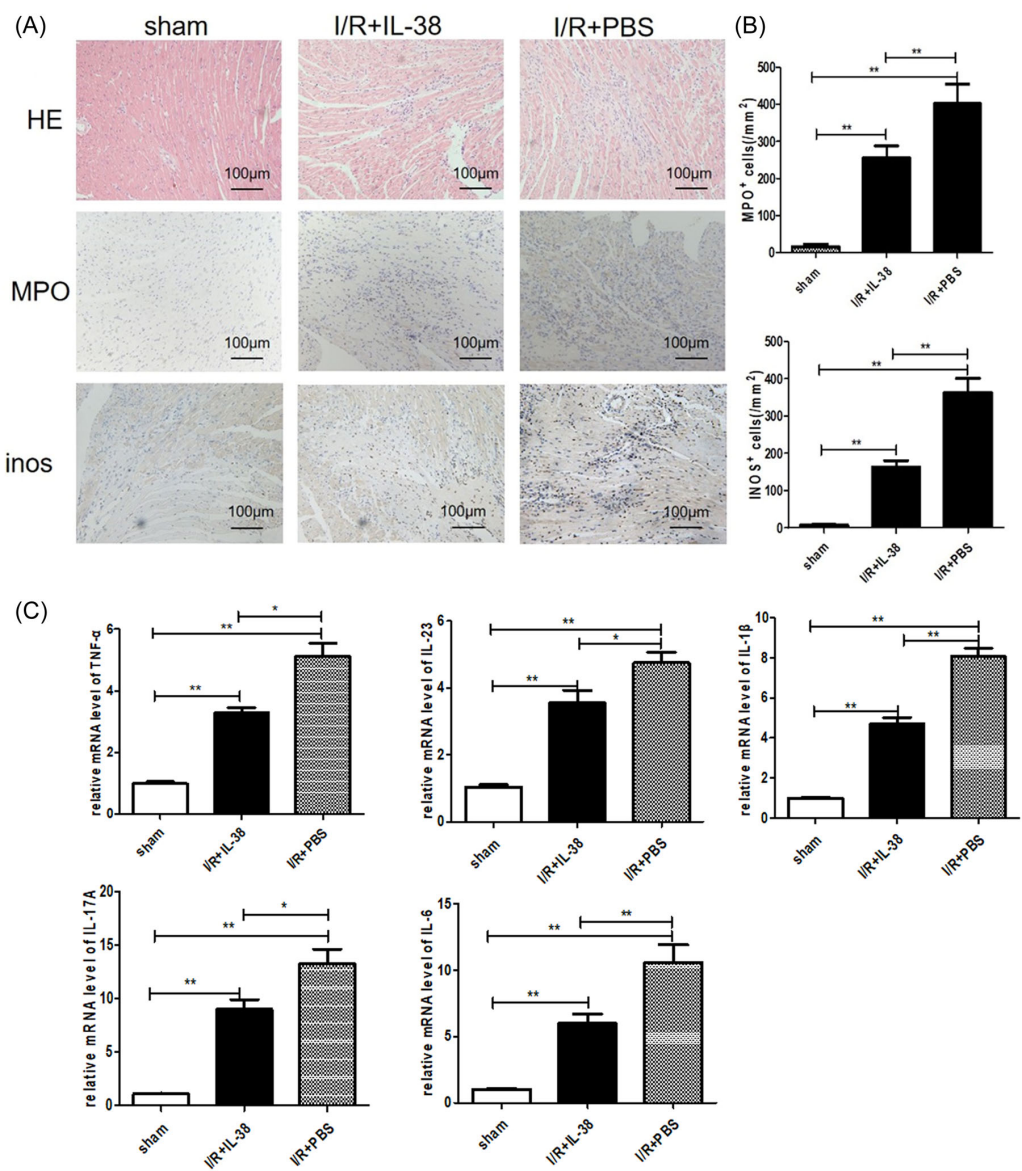
Interleukin‐38 (IL‐38) inhibits inflammatory response in the infarcted heart postreperfusion. (A) Representative images of hematoxylin and eosin (HE) staining, infiltration of myeloperoxidase (MPO^+^) neutrophils and mouse inos^+^ cells in the border area of infarct hearts. Images for HE staining, neutrophils, and INOS^+^ cells are from Day 3 after ischemia/reperfusion (I/R). (B) Infiltration of neutrophils and inos^+^ cells were compared between the different groups at set time points (each group *n* = 6). (C–F) Analysis of messenger RNA (mRNA) levels of tumor necrosis factor‐α (TNF‐α), IL‐23, IL‐1β, IL‐17A, and IL‐6 on Day 3 after I/R. Data are depicted as fold changes versus sham and shown as the mean ± SEM of three to six independent experiments. *p < .05; **p < .01.

Oxygen free radicals and inflammatory cytokines induced by reperfusion play critical roles in tissue damage. When a myocardial injury event occurs, neutrophils, proinflammatory monocytes and lymphocytes are rapidly recruited into the infarcted myocardium to secrete a variety of cytokines that disrupt the immune balance. We next evaluated inflammatory cell infiltration in the injured hearts of different groups of mice. We found that HE^+^ cells, MPO^+^ cells, and INOS^+^ cells were identified less in IL‐38‐treated mice than in PBS‐treated mice after myocardial I/R (Figure 3A,B). The expansion of inflammatory response depends to a certain extent on the secretion of inflammatory factors. The temporal expression level of cytokines in the injured hearts was determined by reverse transcription‐PCR. As shown in Figure 3C–F, consistent with inflammatory cell infiltration, the expression of some inflammatory factors was also inhibited by IL‐38 postreperfusion.

### 3.4 Decreased cardiomyocyte apoptosis in IL‐38‐treated hearts after MIRI

**Figure 4:**
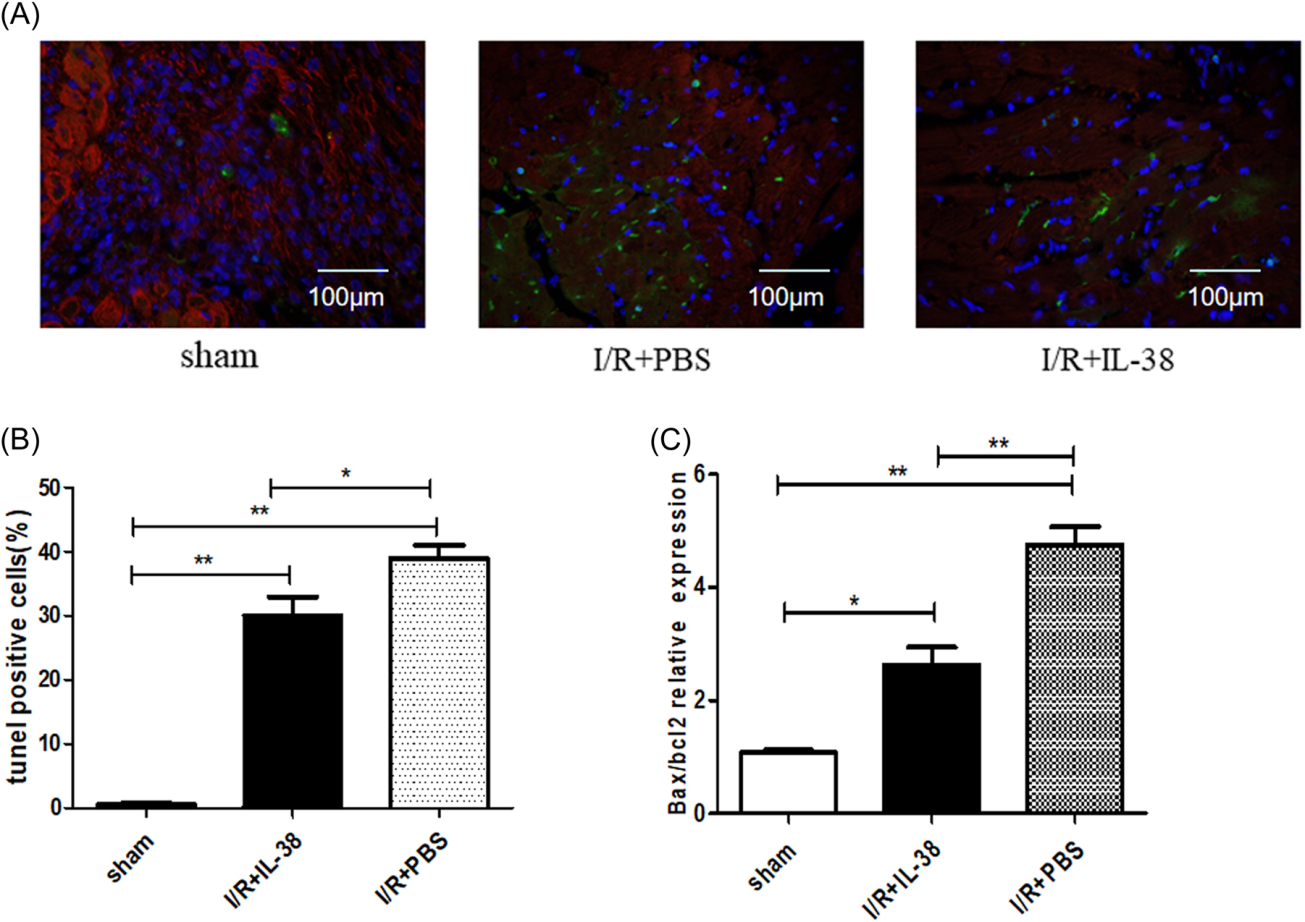
Interleukin‐38 (IL‐38) inhibited cardiomyocyte apoptosis in vivo. (A) Representative images of terminal deoxynucleotidyl transferase dUTP nick‐end labeling (TUNEL)‐stained heart sections from different experimental groups 1 day after ischemia/reperfusion (I/R). TUNEL (green) and 4′,6‐diamidino‐2‐phenylindole (blue) staining of nuclei in apoptotic cardiomyocytes (red) in the peri‐infarct zone. Scar bar:100 μm; magnification: ×400. (B) Quantitative analysis of the percentages of TUNEL‐positive nuclei (each group *n* = 6). (C) Real‐time polymerase chain reaction determined messenger RNA expression levels of Bax and Bcl‐2 in the in infarct heart on Day 1 in I/R. The results were also expressed as the ratio of Bax/Bcl‐2 (*n* = 6). *p < .05; **p < .01.

Cardiac function is mainly maintained by cardiomyocyte contraction and poor ventricular remodeling is closely related to cardiomyocyte apoptosis. As shown in Figure 4A,B, cardiomyocyte apoptosis was significantly increased after myocardial I/R, and most notably, TUNEL‐positive cardiomyocytes decreased after IL‐38 intervention. Additionally, the ratio of Bax/Bcl‐2 was decreased in the injured hearts of MIRI mice treated with rIL‐38 (Figure 4C), suggesting that IL‐38 may be involved in regulating the expression of apoptotic proteins in a certain way, thereby inhibiting the apoptosis of cardiomyocytes.

### 3.5 IL‐38 is released from LPS‐stimulated macrophages and inhibits macrophage cytokine production

**Figure 5:**
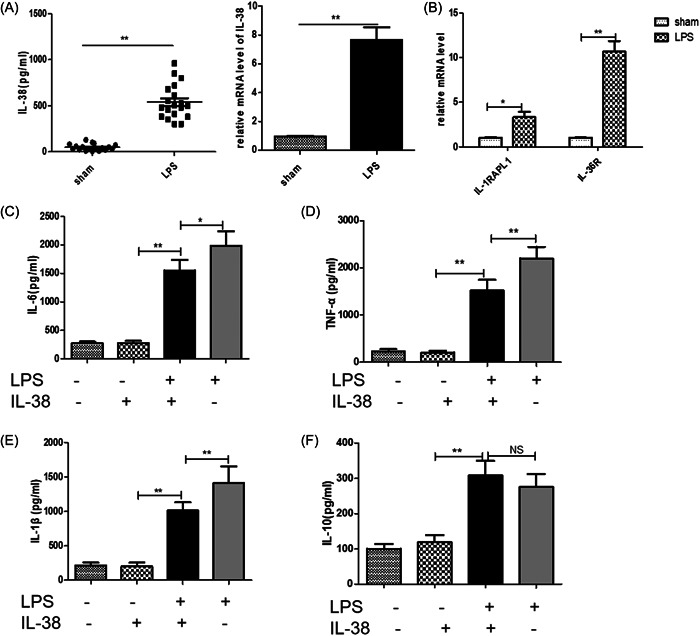
Interleukin‐38 (IL‐38) inhibited lipopolysaccharide (LPS)‐induced macrophage inflammation. Macrophages were incubated with 1 μg/mL LPS and/or 100 ng/mL IL‐38 for 24 h. (A) IL‐38 expression level was assessed by enzyme‐linked immunosorbent assay (ELISA) (in cultured supernatants) and real‐time polymerase chain reaction (PCR) in cultured macrophages. (B) Real‐time PCR determined messenger RNA (mRNA) expression level of IL‐38 receptors. The expression levels of IL‐6 (C), tumor necrosis factor‐α (TNF‐α) (D), IL‐1β (E), and IL‐10 (F) were measured by ELISA in cultured supernatants of different treatment groups (each group *n* = 6–8). *p < .05; **p < .01; NS, not significant.

The results of in vitro experiments suggested that macrophages in the injured hearts of MIRI mice are the main cells producing IL‐38. We then performed further in vitro experiments using RAW264.7 macrophages. As shown in Figure 5A, we detected a significant increase in IL‐38 in the supernatants of LPS‐stimulated macrophages. In addition, we also examined the major receptors of IL‐38. Both IL‐36R and IL‐1R1 were increased after LPS stimulation, but IL‐36R seemed to increase more significantly. We next quantified the level of IL‐6, TNF‐α, IL‐1β, and IL‐10 in the supernatants of different cultured macrophages. As shown in Figure 5C, higher levels of IL‐6, IL‐1β, and TNF‐α were detected in LPS‐ stimulated macrophages compared to LPS and IL‐38‐treated macrophages. However, IL‐10 expression was not statistically significant between the two groups.

### 3.6 IL‐38 inhibits macrophage inflammation in the presence of TnI

**Figure 6:**
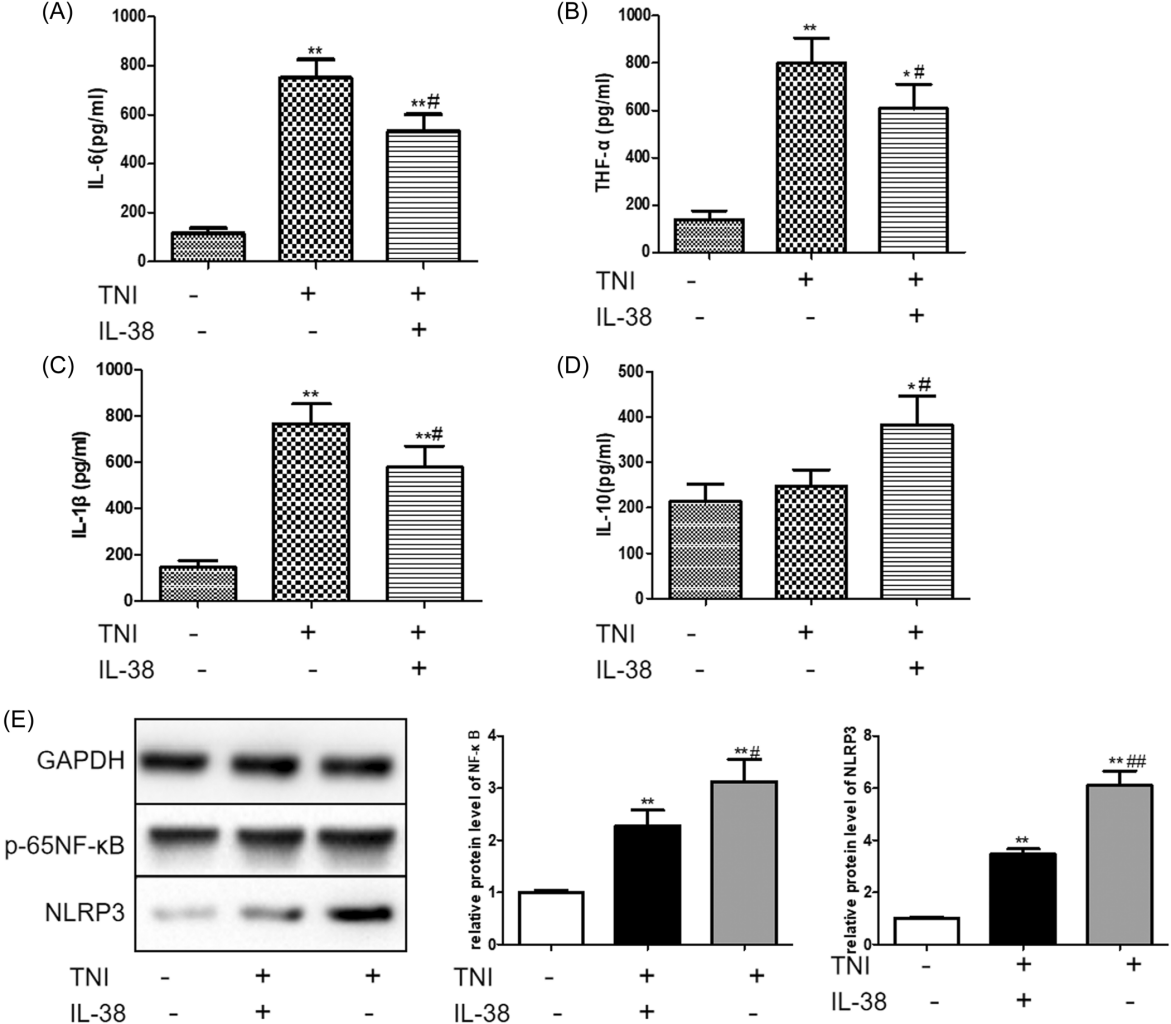
Interleukin‐38 (IL‐38) inhibited macrophage inflammation in the presence of troponin I (TnI). Macrophages were incubated with 10 ng/mL lipopolysaccharide (LPS) in the presence/absence of 1 μg/mL TnI and/or 50 ng/mL IL‐38 for 24 h. The expression levels of IL‐6 (A), tumor necrosis factor‐α (TNF‐α) (B), IL‐1β (C), and IL‐10 (D) were measured by enzyme‐linked immunosorbent assay (ELISA) in cultured supernatants of different treatment groups. (E) Protein was extracted from cultured cardiomyocytes. Representative images of a western blot for expression levels of p65NF‐κB and NOD‐like receptor pyrin domain‐related protein 3 (NLRP3) (*n* = 6). *p < .05 vs. sham (LPS^+^TnI^−^IL‐38^−^), **p < .01 vs. sham (LPS^+^TnI^−^IL‐38^−^); ^#^ *p* < .05 vs. (LPS^+^TnI^+^IL‐38^−^), ^##^ *p* < .01 vs. (LPS^+^TnI^+^IL‐38^−^).

TnI has been considered as a gold‐standard biomarker for acute coronary syndrome with high specificity and sensitivity and is secreted by injured myocardial tissue. To better mimic the microenvironment during reperfusion injury, we examined the effect of IL‐38 in macrophages in the presence of TnI. As shown in Figure 6A–D, IL‐38 stimulation decreased the expression of IL‐6, TNF‐α, and IL‐1β in the supernatants of cultured macrophages. Additionally, IL‐38 stimulation also increased IL‐10 secretion in the presence of TnI.

Activation of the NF‐κB signaling pathway is demonstrated to be closely related to the occurrence of excessive inflammatory response.^20^ We found that IL‐38 inhibited the expression of P65‐NF‐κB in macrophages stimulated with low concentrations of LPS and TnI. In addition, IL‐38 also inhibited the expression of NLRP3, indicating that IL‐38 may mediate the activation of the inflammasome.

### 3.7 IL‐38/TnI‐treated macrophage alleviated cardiomyocyte apoptosis

**Figure 7:**
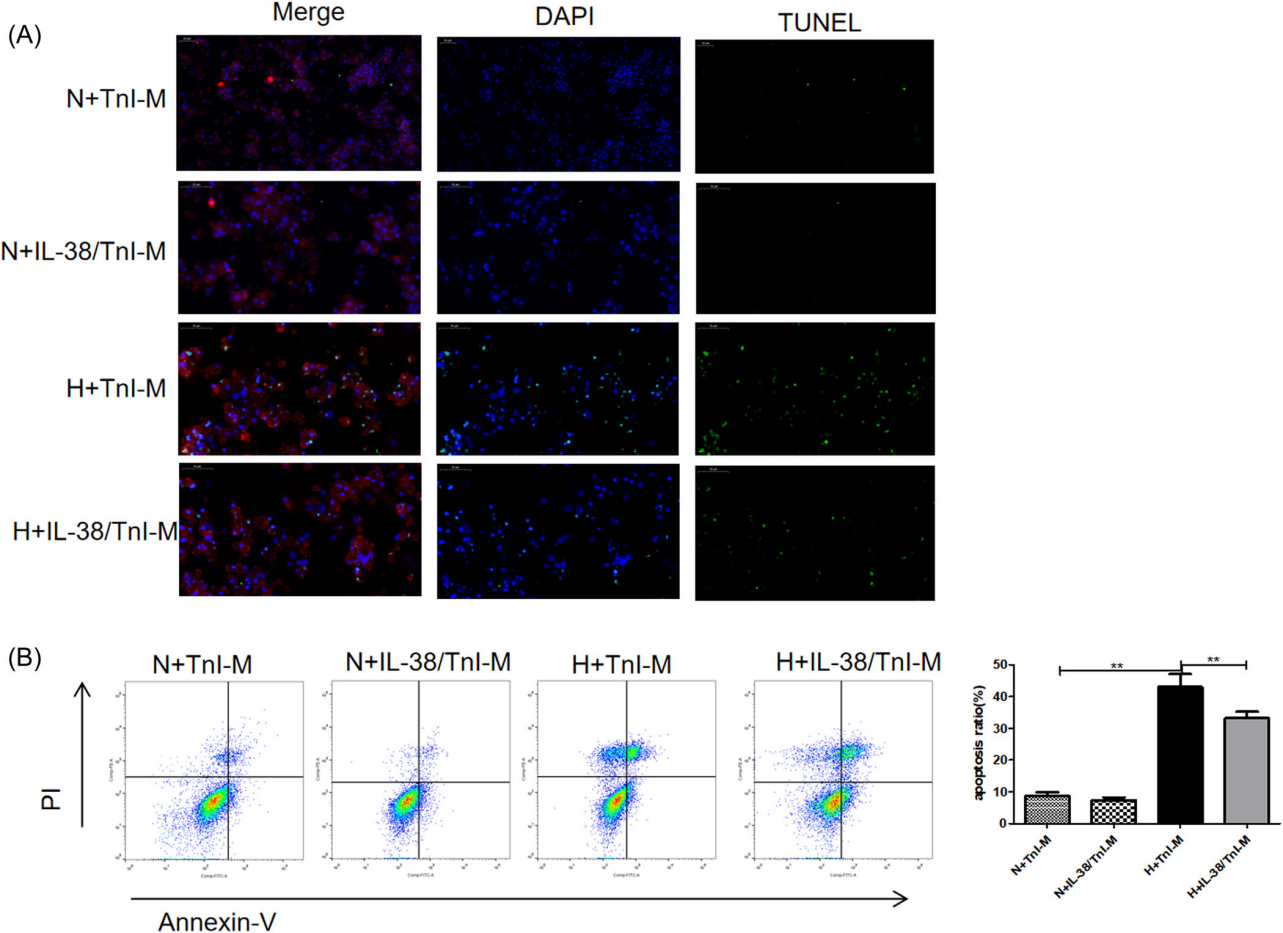
Macrophages induced by interleukin‐38 (IL‐38) and troponin I (TnI) alleviated cardiomyocyte apoptosis. The supernatant of macrophages induced by TnI(TnI‐M) and IL‐38+TnI(IL‐38/TnI‐M) was used for coculture with cardiomyocytes. The mixed cells were cultured in anaerobic conditions and maintained at 37°C for 6 h and then transferred to the normoxic incubator for 6 h to undergo reoxygenation (hypoxia [H]). Cells without anaerobic conditions served as a control (normoxia [N]). (A) Representative images of terminal deoxynucleotidyl transferase dUTP nick‐end labeling (TUNEL)‐stained sections from cultured cardiomyocytes for the indicated times. TUNEL (green) and 4′,6‐diamidino‐2‐phenylindole (blue) staining of nuclei in apoptotic cardiomyocytes (red). Scar bar: 50 μm, magnification: ×400. (B). Representative images and quantitative analysis of apoptotic rate were assessed as (Annexin V (+) PI (−) cells+ Annexin V (+) PI (+) cells)/total cells × 100% using flow cytometry (*n* = 6). **p < .01.

Primary cardiomyocytes were cocultured with the supernatant of macrophages treated by TnI and/or IL‐38, respectively. The cocultured cells were placed in an anaerobic incubator and maintained at 37°C for 6 h to establish a cellular hypoxia stimulation and then were transferred to the normoxic incubator for 6 h to reoxygenation. As shown in Figure 7A,B, cardiomyocytes showed a lower rate of apoptosis when they cocultured with the supernatant of IL‐38‐ and TnI‐stimulated macrophages under the condition of hypoxia and reoxygenation, compared with cocultured with the supernatant of macrophages stimulated by TnI alone.

## 4 DISCUSSION

As a newly identified IL‐1 family cytokine, IL‐38 has been reported to be involved in a variety of inflammatory diseases. Previous studies have shown that the correlation of IL‐38 gene polymorphism and expression levels of serum C‐reactive protein might reflect a role for IL‐38 in systemic inflammatory responses.^12^, ^21^ Notably, the polygenic analysis revealed that rs6734238 in the IL1F10/IL1RN locus was found to be significantly associated with coronary artery disease.^22^ In this study, we used I/R mice and hypoxia/reoxygenation cell models to reveal the apparent role and potential of IL‐38 in MIRI. We found that reperfusion injury induced IL‐38 secretion and rIL‐38 improved ventricular remodeling after reperfusion. The protective effect of IL‐38 in MIRI may be achieved by inhibiting macrophage inflammation, resulting in decreased expression of inflammatory factors and reduced cardiomyocyte apoptosis. Taken together, these findings enriched the theoretical basis for the study of IL‐38 in ischemic cardiomyopathy.

Vascular recanalization is the best therapy to rescue cardiomyocytes after AMI. However, myocardial injury induced by reperfusion therapy has become a severe effect on the postoperative outcomes of patients.^18^, ^23^ The occurrence and progression of MIRI is a complex process in which many genes are involved in the regulation.^24^ Currently, it is generally believed that cardiomyocyte death, including apoptosis, pyrodeath, and necrosis mediated by neutrophil aggregation, immune inflammatory response, calcium overload, and oxygen free radical generation is the main pathogenesis of reperfusion injury.^25^, ^26^ IL‐38 gene polymorphisms have been reported to be associated with many inflammatory IL‐1‐driven diseases by modulating immune inflammatory responses.^9^, ^10^, ^11^ Previous studies have shown that IL‐38 can be released from apoptotic macrophages, A549 lung, and MDA‐231 breast cancer cells.^16^ In the current study, the expression of IL‐38 detected in infarcted heart tissue was mainly secreted by local macrophages and cardiomyocytes. This result appears to be generally consistent with our previous observations in myocardial infarction, although it was found to be abundantly expressed in apoptotic cardiomyocytes after AMI. This may be related to the slightly different timing of inflammatory cell infiltration between AMI and acute myocardial reperfusion injury. Furthermore, rIL‐38 inhibited the secretion of TNF‐α, IL‐6, and IL‐1β by LPS‐induced macrophages in vitro. However, we noticed that it did not increase IL‐10 expression, which may be related to the concentration of IL‐38 or LPS or the degree of macrophage differentiation. These data also suggest that IL‐38, as a factor secreted by macrophages, serves a protective role against myocardial damage.

Cardiac TnI is secreted by injured myocardial tissue and is a gold‐standard biomarker for acute coronary syndrome. When TnI is released into circulation, the previously concealed autoantigen could induce autoimmune responses. A previous study has reported that tolerance induction by nasal vaccination with troponin alleviated I/R injury.^27^ In the present study, we compared the role of IL‐38 on macrophages in the presence of TnI. We found that macrophages cotreated with IL‐38 and TnI secreted less proinflammatory factors than macrophages treated with TnI alone, indicating that IL‐38 may mediate TnI‐induced phenotypic differentiation of macrophages. These results also suggested that IL‐38 may promote the differentiation of macrophages into less inflammatory macrophages under inflammatory conditions, which are commonly called alternatively activated or M2 macrophages. Moreover, TnI‐dependent immune tolerance is expected to provide therapeutic intervention for MIRI.

It is extensively studied and well established that activation of the nuclear factor‐κB (NF‐κB) signaling pathway plays a critical role in the regulation of inflammatory response and apoptosis and is involved in the pathogenesis of cardiac dysfunction.^28^, ^29^, ^30^ The formation of receptor heterodimers can induce biological responses typically involving the activation of MAPK and NF‐κB pathways.^31^ Recent studies have shown that IL‐38 could inhibit the activation of intracellular STAT1/3, ERK1/2, P38 MAPK, and NF‐κB signaling pathways, and upregulate the expression of antiallergic response gene RGS13 and host defense‐related gene POU2AF1 in mice with allergic asthma.^32^ Our results showed that IL‐38 effectively inhibited P65NF‐κB activation in macrophages and interfered with the expression of NLRP3 inflammasome. The NLRP3 inflammasome is a multiprotein complex containing NLRP3, ASC, and caspase‐1.^33^ A growing number of studies suggest that it may play a potential role in ischemic heart disease.^34^ When inflammatory cells infiltrate into the damaged heart, inflammasome activation, and pyroptosis in tissue‐resident cells following MIRI may generate a proinflammatory microenvironment.^35^ Inhibition of NF‐κB and NLRP3 by IL‐38 suggested the priming step of inflammasome activation is mainly mediated by NF‐κB, which is consistent with previous studies.^36^ Therefore, the protective effect of IL‐38 may partly attribute to inhibiting the NF‐κB pathway and mediating inflammasome activation, which further inhibits the expression of IL‐1β and other related factors, leading to mild ventricular remodeling.

IL‐38 has been identified as having similar biological activities to IL‐1 family members because of its homology with other IL‐1F members.^37^ It has been reported that IL‐38 mainly functions as a typical receptor antagonist in inflammation regulation similar to IL‐1Ra and IL‐36Ra, and inhibits the IL‐36 signaling pathway.^38^ Previous studies have shown that truncated IL‐38 reduces ACM‐induced cytokine secretion by attenuating the JNK/AP1 pathway downstream of IL1RAPL1 in macrophages.^16^ We examined the expression of two classical IL‐38 receptors on the surface of macrophages. Both IL‐36R and IL‐1R1 increased after the LPS challenge, but IL‐36R seemed to increase more significantly, which indicates that IL‐38 may bind IL‐36R to inhibit NF‐κB activation and reduce LPS‐induced inflammatory cytokines expression. However, IL‐38 receptors have not been fully identified. Which receptor IL‐38 acts on and whether a second receptor is required when it binds to the IL‐36R or IL‐1R requires further investigation.

The expression changes of IL‐38 in patients with myocardial infarction and reperfusion therapy suggest its possible role in ischemic cardiomyopathy.^12^ The occurrence and progression of MIRI is a complex process regulated by many genes. Cardiomyocyte apoptosis and pyroptosis induced by oxidative stress play critical roles in the pathological progress of worsening ventricular remodeling in various types of heart diseases.^39^ In the present study, IL‐38 attenuated cardiomyocyte apoptosis in infarcted hearts after MIRI. In combination with the results of our previous and current studies, this antiapoptotic effect may be achieved through autocrine and paracrine modes and was largely dependent on the decreased activation of inflammatory factors.

In conclusion, we demonstrated the protective effect of IL‐38 against myocardial I/R injury, which may be partly attributable to inhibiting the activation of inflammatory macrophages, thus decreasing myocardial apoptosis. However, this study has some limitations as follows: (1) the number of our samples is relatively small; (2) the effect of IL‐38 low expression in MIRI mice was not elaborated; (3) the protective role and molecular mechanisms of IL‐38 in ischemic cardiomyopathy have not been thoroughly studied, and we need more in‐depth studies in transgenic mice. Whether IL‐38 should be considered as a potential therapeutic target in ischemic cardiomyopathy remains for further investigation and confirmation.

## AUTHOR CONTRIBUTIONS

**Yuzhen Wei**: Conceptualization (supporting); data curation (lead); formal analysis (lead); writing—original draft (lead); funding acquisition (equal). **Junhui Xing**: Conceptualization (supporting); writing—original draft (supporting); writing—review and editing (supporting). **Xin Su**: Software (lead); methodology (equal); writing—review and editing (supporting). **Xiangrao Li**: Data curation (supporting); funding acquisition (supporting); formal analysis (supporting). **Xiaofei Yan**: Methodology (equal). **Jiangtao Zhao**: writing—review and editing (supporting). **Hailong Tao**: Conceptualization (lead); supervision (lead), funding acquisition (equal); writing—review and editing (supporting); project administration (lead).

## CONFLICT OF INTEREST STATEMENT

The authors declare no conflict of interest.

## Data Availability

Article data are available by mail from the corresponding author
